# (*E*)-*N*′-(5-Bromo-2-meth­oxy­benzyl­idene)isonicotinohydrazide

**DOI:** 10.1107/S1600536811017910

**Published:** 2011-05-20

**Authors:** Hong-Yan Ban

**Affiliations:** a School of Chemical Engineering, University of Science and Technology Liaoning, Anshan 114051, People’s Republic of China

## Abstract

The asymmetric unit of the title compound, C_14_H_12_BrN_3_O_2_, contains two independent mol­ecules in which the dihedral angles between the benzene ring and the pyridine ring are 24.4 (6) and 23.7 (6)°. The mol­ecules exist in a *trans* configuration with respect to the central methyl­idene units. In the crystal, mol­ecules are linked through inter­molecular N—H⋯O hydrogen bonds, forming chains along the *a* axis.

## Related literature

For the biological activity of hydrazones, see: Zhong *et al.* (2007 ▶); Raj *et al.* (2007 ▶); Jimenez-Pulido *et al.* (2008 ▶). For related structures, see: Ban (2010 ▶); Ban & Li (2008*a* ▶,*b* ▶); Li & Ban (2009*a* ▶,*b* ▶); Yehye *et al.* (2008 ▶); Fun *et al.* (2008*a* ▶,*b* ▶); Yang *et al.* (2008 ▶); Ejsmont *et al.* (2008 ▶); Yang (2006 ▶).
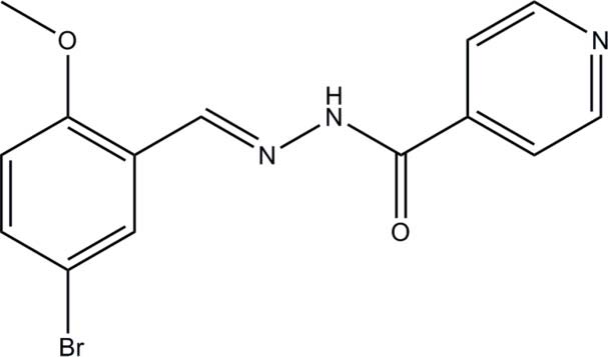


## Experimental

### 

#### Crystal data

C_14_H_12_BrN_3_O_2_
*M* *_r_* = 334.18
Monoclinic, 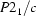

*a* = 10.020 (3) Å
*b* = 25.732 (2) Å
*c* = 11.243 (2) Å
β = 102.199 (3)°
*V* = 2833.4 (10) Å^3^
*Z* = 8
Mo *K*α radiation
μ = 2.91 mm^−1^
*T* = 298 K
0.13 × 0.10 × 0.10 mm

#### Data collection

Bruker SMART CCD area-detector diffractometer
Absorption correction: multi-scan (*SADABS*; Sheldrick, 1996 ▶) *T*_min_ = 0.704, *T*_max_ = 0.760
14099 measured reflections
5922 independent reflections
2018 reflections with *I* > 2σ(*I*)
*R*_int_ = 0.114

#### Refinement

*R*[*F* ^2^ > 2σ(*F* ^2^)] = 0.064
*wR*(*F* ^2^) = 0.143
*S* = 0.94
5922 reflections
369 parameters
2 restraints
H atoms treated by a mixture of independent and constrained refinement
Δρ_max_ = 0.32 e Å^−3^
Δρ_min_ = −0.43 e Å^−3^

### 

Data collection: *SMART* (Bruker, 1998 ▶); cell refinement: *SAINT* (Bruker, 1998 ▶); data reduction: *SAINT*; program(s) used to solve structure: *SHELXS97* (Sheldrick, 2008 ▶); program(s) used to refine structure: *SHELXL97* (Sheldrick, 2008 ▶); molecular graphics: *SHELXTL* (Sheldrick, 2008 ▶); software used to prepare material for publication: *SHELXTL*.

## Supplementary Material

Crystal structure: contains datablocks global, I. DOI: 10.1107/S1600536811017910/sj5145sup1.cif

Structure factors: contains datablocks I. DOI: 10.1107/S1600536811017910/sj5145Isup2.hkl

Supplementary material file. DOI: 10.1107/S1600536811017910/sj5145Isup3.cml

Additional supplementary materials:  crystallographic information; 3D view; checkCIF report

## Figures and Tables

**Table 1:** Hydrogen-bond geometry (Å, °)

*D*—H⋯*A*	*D*—H	H⋯*A*	*D*⋯*A*	*D*—H⋯*A*
N5—H5⋯O2	0.90 (1)	2.06 (2)	2.939 (7)	164 (6)
N2—H2⋯O4^i^	0.90 (1)	2.11 (2)	2.999 (7)	168 (6)

Symmetry code: (i) 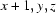.

**Fig. 1.**
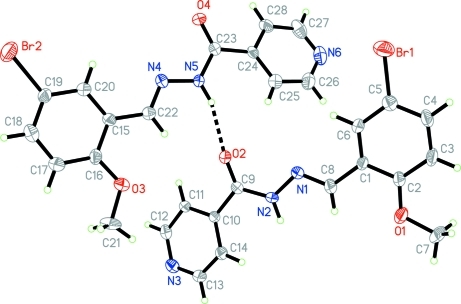
The molecular structure of the title compound, showing 30% probability displacement ellipsoids for the non-hydrogen atoms. A hydrogen bond is shown as a dashed line.

**Fig. 2.**
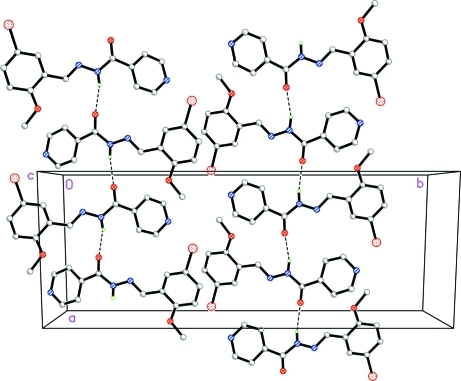
The packing diagram of the title compound, viewed along the c axis. Hydrogen bonds are shown as dashed lines.

## supplementary crystallographic information

### Comment

Hydrazone compounds derived from the condensation of aldehydes with hydrazides have been demonstrated to possess excellent biological activities (Zhong *et al.*, 2007; Raj *et al.*, 2007; Jimenez-Pulido *et al.*, 2008). Due to the easy synthesis of such compounds, a number of hydrazone compounds have been synthesized and structurally characterized (Yehye *et al.*, 2008; Fun *et al.*, 2008*a*,*b*; Yang *et al.*, 2008; Ejsmont *et al.*, 2008; Yang, 2006). Recently, we have reported a few such compounds (Ban, 2010; Ban & Li, 2008*a*,*b*; Li & Ban, 2009*a*,*b*). We report here the crystal structure of the title new compound.

The asymmetric unit of the title hydrazone compound, Fig. 1, contains two independent molecules. The dihedral angles between the benzene ring and the pyridine ring in each molecule are  24.4 (6) and 23.7 (6)°, respectively. The molecules exist in a *trans* configuration with respect to the central methylidene units. In the crystal structure, molecules are linked through intermolecular N—H···O hydrogen bonds (Table 1), forming chains along the *a* axis, Fig. 2.

### Experimental

The title compound was prepared by refluxing 5-bromo-2-methoxybenzaldehyde (1.0 mol, 0.22 g) with isonicotinohydrazide (1.0 mol, 0.14 g) in methanol (100 ml). Excess methanol was removed from the mixture by distillation. The colourless solid product was filtered, and washed three times with methanol. Colourless block-shaped crystals of the title compound were obtained from a methanol solution of the compound by slow evaporation in air.

### Refinement

Atoms H2 and H5 were located in a difference Fourier map and refined isotropically, with the N—H distances restrained to 0.90 (1) Å. The remaining H atoms were placed in calculated positions (C—H = 0.93–0.96 Å) and refined as riding with *U*_iso_(H) = 1.2*U*_eq_(C) and 1.5 *U*_eq_(C7 and C21).

### Crystal data

**Table:**

C_14_H_12_BrN_3_O_2_	*F*(000) = 1344
*M**_r_* = 334.18	*D*_x_ = 1.567 Mg m^−^^3^
Monoclinic, *P*2_1_/*c*	Mo *K*α radiation, λ = 0.71073 Å
Hall symbol: -P 2ybc	Cell parameters from 916 reflections
*a* = 10.020 (3) Å	θ = 2.5–24.3°
*b* = 25.732 (2) Å	µ = 2.91 mm^−^^1^
*c* = 11.243 (2) Å	*T* = 298 K
β = 102.199 (3)°	Block, colourless
*V* = 2833.4 (10) Å^3^	0.13 × 0.10 × 0.10 mm
*Z* = 8	

### Data collection

**Table:**

Bruker SMART CCD area-detector diffractometer	5922 independent reflections
Radiation source: fine-focus sealed tube	2018 reflections with *I* > 2σ(*I*)
graphite	*R*_int_ = 0.114
ω scans	θ_max_ = 27.0°, θ_min_ = 2.0°
Absorption correction: multi-scan (*SADABS*; Sheldrick, 1996)	*h* = −12→12
*T*_min_ = 0.704, *T*_max_ = 0.760	*k* = −15→32
14099 measured reflections	*l* = −12→14

### Refinement

**Table:**

Refinement on *F*^2^	Primary atom site location: structure-invariant direct methods
Least-squares matrix: full	Secondary atom site location: difference Fourier map
*R*[*F*^2^ > 2σ(*F*^2^)] = 0.064	Hydrogen site location: inferred from neighbouring sites
*wR*(*F*^2^) = 0.143	H atoms treated by a mixture of independent and constrained refinement
*S* = 0.94	*w* = 1/[σ^2^(*F*_o_^2^)] where *P* = (*F*_o_^2^ + 2*F*_c_^2^)/3
5922 reflections	(Δ/σ)_max_ < 0.001
369 parameters	Δρ_max_ = 0.32 e Å^−^^3^
2 restraints	Δρ_min_ = −0.43 e Å^−^^3^

### Special details

**Table:**

Geometry. All e.s.d.'s (except the e.s.d. in the dihedral angle between two l.s. planes) are estimated using the full covariance matrix. The cell e.s.d.'s are taken into account individually in the estimation of e.s.d.'s in distances, angles and torsion angles; correlations between e.s.d.'s in cell parameters are only used when they are defined by crystal symmetry. An approximate (isotropic) treatment of cell e.s.d.'s is used for estimating e.s.d.'s involving l.s. planes.
Refinement. Refinement of *F*^2^ against ALL reflections. The weighted *R*-factor *wR* and goodness of fit *S* are based on *F*^2^, conventional *R*-factors *R* are based on *F*, with *F* set to zero for negative *F*^2^. The threshold expression of *F*^2^ > σ(*F*^2^) is used only for calculating *R*-factors(gt) *etc*. and is not relevant to the choice of reflections for refinement. *R*-factors based on *F*^2^ are statistically about twice as large as those based on *F*, and *R*- factors based on ALL data will be even larger.

### Fractional atomic coordinates and isotropic or equivalent isotropic displacement parameters (Å^2^)

**Table:**

	*x*	*y*	*z*	*U*_iso_*/*U*_eq_	
Br1	0.51353 (8)	0.35943 (3)	0.41403 (8)	0.0813 (3)	
Br2	0.03344 (8)	−0.09381 (3)	0.49305 (8)	0.0786 (3)	
N1	0.7533 (5)	0.1886 (2)	0.2601 (5)	0.0433 (14)	
N2	0.7920 (5)	0.1410 (2)	0.2216 (5)	0.0483 (15)	
N3	0.8554 (7)	−0.0399 (2)	0.0738 (5)	0.0570 (17)	
N4	0.2636 (5)	0.0667 (2)	0.2875 (5)	0.0469 (15)	
N5	0.3008 (5)	0.1126 (2)	0.2381 (5)	0.0455 (15)	
N6	0.3357 (8)	0.2873 (2)	0.0375 (6)	0.070 (2)	
O1	1.0434 (5)	0.29599 (17)	0.2924 (4)	0.0661 (14)	
O2	0.5883 (5)	0.10295 (15)	0.2207 (4)	0.0531 (13)	
O3	0.5607 (5)	−0.03865 (16)	0.3491 (4)	0.0627 (14)	
O4	0.0906 (5)	0.14763 (16)	0.2208 (5)	0.0675 (15)	
C1	0.8234 (7)	0.2757 (2)	0.3160 (6)	0.0396 (17)	
C2	0.9269 (8)	0.3125 (3)	0.3234 (6)	0.0504 (19)	
C3	0.9065 (7)	0.3632 (3)	0.3589 (6)	0.056 (2)	
H3	0.9754	0.3878	0.3643	0.068*	
C4	0.7825 (8)	0.3763 (3)	0.3859 (6)	0.056 (2)	
H4	0.7671	0.4102	0.4087	0.068*	
C5	0.6821 (7)	0.3396 (3)	0.3791 (6)	0.0478 (19)	
C6	0.7036 (7)	0.2897 (3)	0.3450 (6)	0.0497 (19)	
H6	0.6350	0.2651	0.3417	0.060*	
C7	1.1577 (7)	0.3308 (3)	0.3069 (6)	0.070 (2)	
H7A	1.1368	0.3586	0.2489	0.105*	
H7B	1.2364	0.3123	0.2936	0.105*	
H7C	1.1763	0.3449	0.3878	0.105*	
C8	0.8473 (7)	0.2231 (2)	0.2755 (6)	0.054 (2)	
H8	0.9325	0.2146	0.2606	0.064*	
C9	0.7057 (7)	0.1007 (2)	0.2057 (6)	0.0432 (18)	
C10	0.7624 (7)	0.0518 (2)	0.1629 (6)	0.0387 (17)	
C11	0.6714 (7)	0.0151 (2)	0.1033 (6)	0.052 (2)	
H11	0.5777	0.0201	0.0927	0.062*	
C12	0.7233 (8)	−0.0294 (3)	0.0596 (6)	0.058 (2)	
H12	0.6615	−0.0534	0.0177	0.069*	
C13	0.9394 (7)	−0.0048 (3)	0.1323 (6)	0.055 (2)	
H13	1.0325	−0.0113	0.1443	0.066*	
C14	0.8989 (7)	0.0409 (2)	0.1769 (6)	0.0464 (18)	
H14	0.9638	0.0644	0.2165	0.056*	
C15	0.3380 (7)	−0.0171 (2)	0.3649 (6)	0.0431 (18)	
C16	0.4437 (7)	−0.0535 (3)	0.3829 (6)	0.0456 (18)	
C17	0.4289 (7)	−0.1015 (3)	0.4355 (6)	0.055 (2)	
H17	0.4998	−0.1255	0.4484	0.066*	
C18	0.3064 (8)	−0.1126 (3)	0.4681 (6)	0.057 (2)	
H18	0.2953	−0.1445	0.5040	0.068*	
C19	0.2015 (6)	−0.0777 (3)	0.4487 (6)	0.0452 (18)	
C20	0.2170 (7)	−0.0301 (3)	0.3968 (6)	0.0473 (18)	
H20	0.1450	−0.0065	0.3832	0.057*	
C21	0.6772 (7)	−0.0721 (3)	0.3758 (6)	0.072 (2)	
H21A	0.6994	−0.0793	0.4615	0.107*	
H21B	0.7534	−0.0553	0.3525	0.107*	
H21C	0.6571	−0.1040	0.3315	0.107*	
C22	0.3574 (7)	0.0332 (3)	0.3104 (6)	0.0483 (19)	
H22	0.4416	0.0407	0.2919	0.058*	
C23	0.2085 (8)	0.1511 (2)	0.2091 (6)	0.0479 (19)	
C24	0.2575 (7)	0.1983 (2)	0.1524 (6)	0.0398 (18)	
C25	0.3935 (7)	0.2111 (3)	0.1626 (6)	0.059 (2)	
H25	0.4620	0.1904	0.2077	0.071*	
C26	0.4257 (8)	0.2552 (3)	0.1049 (7)	0.069 (2)	
H26	0.5177	0.2632	0.1137	0.083*	
C27	0.2071 (10)	0.2744 (3)	0.0269 (7)	0.073 (3)	
H27	0.1409	0.2951	−0.0214	0.087*	
C28	0.1643 (7)	0.2316 (3)	0.0838 (6)	0.053 (2)	
H28	0.0714	0.2253	0.0755	0.064*	
H5	0.388 (2)	0.116 (2)	0.232 (6)	0.080*	
H2	0.880 (2)	0.139 (3)	0.214 (6)	0.080*	

### Atomic displacement parameters (Å^2^)

**Table:**

	*U*^11^	*U*^22^	*U*^33^	*U*^12^	*U*^13^	*U*^23^
Br1	0.0673 (6)	0.0816 (6)	0.0990 (7)	0.0128 (5)	0.0266 (5)	−0.0272 (6)
Br2	0.0568 (6)	0.0881 (7)	0.0920 (7)	−0.0143 (5)	0.0184 (5)	0.0230 (6)
N1	0.038 (4)	0.035 (3)	0.059 (4)	0.002 (3)	0.016 (3)	−0.003 (3)
N2	0.032 (3)	0.034 (3)	0.081 (4)	0.007 (3)	0.018 (3)	−0.001 (3)
N3	0.057 (4)	0.039 (4)	0.078 (5)	0.007 (4)	0.021 (4)	−0.002 (3)
N4	0.047 (4)	0.032 (3)	0.064 (4)	0.000 (3)	0.016 (3)	−0.002 (3)
N5	0.037 (4)	0.030 (3)	0.074 (4)	−0.001 (3)	0.022 (3)	0.007 (3)
N6	0.091 (6)	0.044 (4)	0.076 (5)	−0.012 (4)	0.021 (5)	0.000 (4)
O1	0.050 (3)	0.054 (3)	0.102 (4)	−0.018 (3)	0.032 (3)	−0.016 (3)
O2	0.042 (3)	0.033 (3)	0.090 (4)	−0.002 (2)	0.026 (3)	0.000 (2)
O3	0.050 (3)	0.049 (3)	0.096 (4)	0.013 (3)	0.028 (3)	0.011 (3)
O4	0.036 (3)	0.045 (3)	0.127 (5)	0.002 (3)	0.032 (3)	0.003 (3)
C1	0.038 (4)	0.034 (4)	0.051 (5)	−0.009 (4)	0.018 (4)	−0.004 (4)
C2	0.049 (5)	0.035 (5)	0.069 (6)	−0.007 (4)	0.018 (4)	−0.012 (4)
C3	0.058 (5)	0.040 (5)	0.074 (6)	−0.021 (4)	0.022 (4)	−0.002 (4)
C4	0.070 (6)	0.033 (4)	0.070 (6)	−0.007 (4)	0.022 (5)	−0.015 (4)
C5	0.051 (5)	0.047 (5)	0.044 (5)	0.002 (4)	0.006 (4)	0.001 (4)
C6	0.039 (5)	0.039 (5)	0.072 (6)	−0.008 (4)	0.014 (4)	−0.008 (4)
C7	0.043 (5)	0.078 (6)	0.094 (6)	−0.020 (4)	0.029 (4)	0.000 (5)
C8	0.053 (5)	0.034 (4)	0.077 (6)	−0.004 (4)	0.021 (4)	−0.011 (4)
C9	0.039 (5)	0.033 (4)	0.059 (5)	0.004 (4)	0.013 (4)	0.003 (4)
C10	0.035 (4)	0.031 (4)	0.055 (5)	0.003 (3)	0.020 (4)	0.007 (4)
C11	0.035 (4)	0.030 (4)	0.090 (6)	0.001 (4)	0.015 (4)	−0.004 (4)
C12	0.058 (6)	0.033 (5)	0.083 (6)	−0.004 (4)	0.018 (5)	0.000 (4)
C13	0.042 (5)	0.043 (5)	0.083 (6)	0.006 (4)	0.018 (4)	−0.003 (5)
C14	0.045 (5)	0.033 (4)	0.063 (5)	−0.002 (4)	0.017 (4)	−0.009 (4)
C15	0.040 (5)	0.033 (4)	0.058 (5)	0.003 (4)	0.015 (4)	−0.008 (4)
C16	0.053 (5)	0.034 (4)	0.053 (5)	−0.009 (4)	0.018 (4)	−0.005 (4)
C17	0.047 (5)	0.050 (5)	0.069 (6)	0.009 (4)	0.013 (4)	0.000 (4)
C18	0.067 (6)	0.042 (5)	0.058 (5)	0.000 (4)	0.008 (4)	0.003 (4)
C19	0.034 (4)	0.045 (5)	0.054 (5)	−0.005 (4)	0.004 (4)	0.006 (4)
C20	0.042 (5)	0.043 (5)	0.059 (5)	0.003 (4)	0.017 (4)	0.000 (4)
C21	0.053 (5)	0.079 (6)	0.085 (6)	0.033 (5)	0.020 (4)	−0.002 (5)
C22	0.046 (5)	0.041 (5)	0.063 (5)	0.001 (4)	0.023 (4)	0.000 (4)
C23	0.045 (5)	0.033 (5)	0.069 (5)	0.001 (4)	0.021 (4)	0.002 (4)
C24	0.039 (4)	0.028 (4)	0.052 (5)	0.000 (4)	0.008 (4)	−0.013 (4)
C25	0.052 (5)	0.048 (5)	0.078 (6)	−0.002 (4)	0.016 (4)	0.000 (5)
C26	0.062 (6)	0.052 (5)	0.092 (7)	−0.010 (5)	0.012 (5)	0.011 (5)
C27	0.106 (8)	0.046 (6)	0.063 (6)	0.013 (5)	0.011 (6)	0.015 (5)
C28	0.045 (5)	0.045 (5)	0.066 (5)	0.011 (4)	0.004 (4)	0.005 (4)

### Geometric parameters (Å, °)

**Table:**

Br1—C5	1.884 (7)	C8—H8	0.9300
Br2—C19	1.900 (6)	C9—C10	1.500 (8)
N1—C8	1.279 (7)	C10—C14	1.373 (8)
N1—N2	1.382 (6)	C10—C11	1.384 (8)
N2—C9	1.337 (8)	C11—C12	1.390 (8)
N2—H2	0.900 (10)	C11—H11	0.9300
N3—C13	1.313 (8)	C12—H12	0.9300
N3—C12	1.326 (8)	C13—C14	1.372 (8)
N4—C22	1.262 (7)	C13—H13	0.9300
N4—N5	1.388 (6)	C14—H14	0.9300
N5—C23	1.347 (8)	C15—C20	1.376 (8)
N5—H5	0.901 (10)	C15—C16	1.395 (8)
N6—C27	1.312 (9)	C15—C22	1.464 (8)
N6—C26	1.335 (8)	C16—C17	1.391 (8)
O1—C2	1.354 (8)	C17—C18	1.384 (9)
O1—C7	1.437 (7)	C17—H17	0.9300
O2—C9	1.224 (7)	C18—C19	1.364 (8)
O3—C16	1.361 (7)	C18—H18	0.9300
O3—C21	1.430 (7)	C19—C20	1.381 (8)
O4—C23	1.219 (7)	C20—H20	0.9300
C1—C6	1.358 (8)	C21—H21A	0.9600
C1—C2	1.394 (8)	C21—H21B	0.9600
C1—C8	1.463 (8)	C21—H21C	0.9600
C2—C3	1.392 (8)	C22—H22	0.9300
C3—C4	1.382 (9)	C23—C24	1.502 (8)
C3—H3	0.9300	C24—C28	1.377 (8)
C4—C5	1.371 (8)	C24—C25	1.383 (8)
C4—H4	0.9300	C25—C26	1.378 (9)
C5—C6	1.369 (8)	C25—H25	0.9300
C6—H6	0.9300	C26—H26	0.9300
C7—H7A	0.9600	C27—C28	1.386 (9)
C7—H7B	0.9600	C27—H27	0.9300
C7—H7C	0.9600	C28—H28	0.9300
			
C8—N1—N2	114.2 (5)	N3—C13—C14	124.4 (7)
C9—N2—N1	120.9 (5)	N3—C13—H13	117.8
C9—N2—H2	124 (4)	C14—C13—H13	117.8
N1—N2—H2	115 (4)	C13—C14—C10	119.9 (6)
C13—N3—C12	116.0 (6)	C13—C14—H14	120.1
C22—N4—N5	114.3 (6)	C10—C14—H14	120.1
C23—N5—N4	119.6 (6)	C20—C15—C16	118.9 (6)
C23—N5—H5	122 (4)	C20—C15—C22	121.7 (6)
N4—N5—H5	118 (4)	C16—C15—C22	119.3 (6)
C27—N6—C26	115.3 (7)	O3—C16—C17	122.9 (6)
C2—O1—C7	119.0 (5)	O3—C16—C15	116.4 (6)
C16—O3—C21	119.1 (5)	C17—C16—C15	120.7 (7)
C6—C1—C2	119.3 (6)	C18—C17—C16	118.5 (7)
C6—C1—C8	122.2 (6)	C18—C17—H17	120.7
C2—C1—C8	118.6 (6)	C16—C17—H17	120.7
O1—C2—C3	123.7 (6)	C19—C18—C17	121.1 (7)
O1—C2—C1	116.3 (6)	C19—C18—H18	119.4
C3—C2—C1	120.1 (7)	C17—C18—H18	119.4
C4—C3—C2	119.1 (6)	C18—C19—C20	120.0 (6)
C4—C3—H3	120.4	C18—C19—Br2	120.7 (6)
C2—C3—H3	120.4	C20—C19—Br2	119.3 (5)
C5—C4—C3	120.1 (6)	C15—C20—C19	120.6 (6)
C5—C4—H4	120.0	C15—C20—H20	119.7
C3—C4—H4	120.0	C19—C20—H20	119.7
C6—C5—C4	120.4 (6)	O3—C21—H21A	109.5
C6—C5—Br1	120.9 (6)	O3—C21—H21B	109.5
C4—C5—Br1	118.7 (6)	H21A—C21—H21B	109.5
C1—C6—C5	121.1 (6)	O3—C21—H21C	109.5
C1—C6—H6	119.5	H21A—C21—H21C	109.5
C5—C6—H6	119.5	H21B—C21—H21C	109.5
O1—C7—H7A	109.5	N4—C22—C15	122.1 (6)
O1—C7—H7B	109.5	N4—C22—H22	119.0
H7A—C7—H7B	109.5	C15—C22—H22	119.0
O1—C7—H7C	109.5	O4—C23—N5	123.5 (6)
H7A—C7—H7C	109.5	O4—C23—C24	121.0 (6)
H7B—C7—H7C	109.5	N5—C23—C24	115.4 (6)
N1—C8—C1	121.3 (6)	C28—C24—C25	115.9 (6)
N1—C8—H8	119.4	C28—C24—C23	119.8 (6)
C1—C8—H8	119.4	C25—C24—C23	124.3 (6)
O2—C9—N2	123.9 (6)	C26—C25—C24	118.8 (7)
O2—C9—C10	121.6 (6)	C26—C25—H25	120.6
N2—C9—C10	114.5 (6)	C24—C25—H25	120.6
C14—C10—C11	117.0 (6)	N6—C26—C25	125.5 (8)
C14—C10—C9	124.8 (6)	N6—C26—H26	117.3
C11—C10—C9	118.2 (6)	C25—C26—H26	117.3
C10—C11—C12	118.4 (6)	N6—C27—C28	123.6 (7)
C10—C11—H11	120.8	N6—C27—H27	118.2
C12—C11—H11	120.8	C28—C27—H27	118.2
N3—C12—C11	124.3 (7)	C24—C28—C27	120.9 (7)
N3—C12—H12	117.9	C24—C28—H28	119.5
C11—C12—H12	117.9	C27—C28—H28	119.5

### Hydrogen-bond geometry (Å, °)

**Table:**

*D*—H···*A*	*D*—H	H···*A*	*D*···*A*	*D*—H···*A*
N5—H5···O2	0.90 (1)	2.06 (2)	2.939 (7)	164 (6)
N2—H2···O4^i^	0.90 (1)	2.11 (2)	2.999 (7)	168 (6)

Symmetry codes: (i) *x*+1, *y*, *z*.
